# Intermetatarsal Coalition: Case Report, Literature Review, and Imaging Findings of an Underrecognized and Underdiagnosed Cause of Foot Pain

**DOI:** 10.1155/2021/6667907

**Published:** 2021-02-08

**Authors:** WanYin Lim, Steven Zadow, Angela Moran, Jonathan Heysen

**Affiliations:** ^1^Dr. Jones and Partners, 226 Greenhill Rd., Eastwood SA5063, Australia; ^2^Royal Adelaide Hospital, 1 Port Rd., Adelaide SA5000, Australia; ^3^Flinders Medical Centre, Flinders Dr., Bedford Park SA5042, Australia; ^4^Sportsmed Stepney Healthcare Hub, 32 Payneham Rd., Stepney SA5069, Australia

## Abstract

Coalition is defined as abnormal bridging between two bones, and the connection can be osseous or nonosseous. Most coalitions in the foot involve the hindfoot. Intermetatarsal coalition is thought to be much rarer than the more common hindfoot coalitions and has only been reported sporadically in the orthopedic literature. We present two patients with nonosseous intermetatarsal coalition presenting with nonspecific dorsolateral foot pain and describe the imaging findings of intermetatarsal coalition with different modalities. We suspect that whilst rarely described, intermetatarsal coalition is quite likely a more common underrecognized entity than a rare entity. This report is aimed at increasing the awareness of coalition in this location, in the radiology community, particularly the nonosseous ones, given that this condition can be debilitating but treatable.

## 1. Introduction

Coalition, or abnormal bridging between two bones, has a prevalence of up to 13% in the foot with majority of the foot coalition occurring in the hindfoot [1, 2]. Intermetatarsal coalition, on the other hand, is much rarer and has only been described in a small number of orthopedic papers [2–10]. We suspect that whilst rarely described, intermetatarsal coalition is likely a more common but underrecognized entity rather than a rare entity. This report is aimed at increasing the awareness of intermetatarsal coalition by describing imaging findings utilising various imaging modalities, with attention given to nonosseous intermetatarsal coalition.

## 2. Case Presentation

### 2.1. Case 1

**Figure 1:**
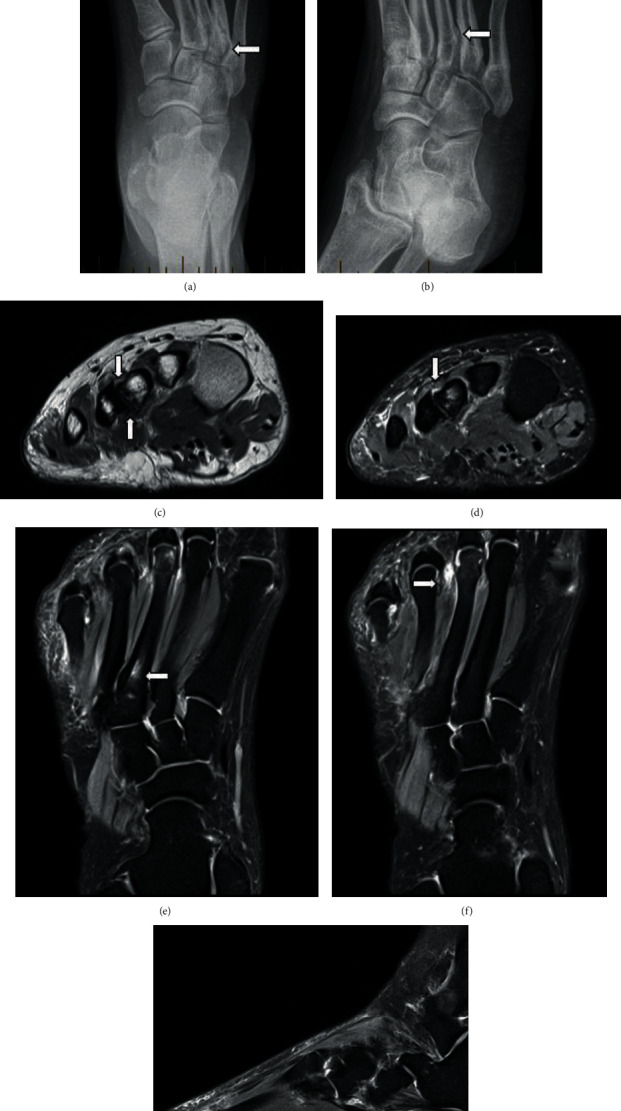
40-year-old female with dorsal forefoot pain following a change in activity. AP (a) and oblique (b) radiographs reveal bony prominence of the lateral border of the proximal third metatarsal shaft. Short axis PD (c) and PDFS (d) sequences reveal abnormal articulation between the base of the third and fourth metatarsals. Surrounding reactive bony hypertrophy and intramedullary marrow oedema signal. This is consistent with nonosseous intermetatarsal coalition. Coronal PDFS (e) sequence with nonosseous coalition between the base of the third and fourth metatarsals. Localized marrow oedema signal at the third metatarsal base. Coronal PDFS (f) sequence reveals a bursal-neuroma complex at the third intermetatarsal space corresponding to the location of the coalition. Sagittal PDFS sequences (g) with low-grade marrow oedema in the third metatarsal head. These are speculated to arise from the altered weight-bearing mechanics as a result of the coalition with increased load on the metatarsal head.

A 40-year-old otherwise healthy female had an MRI and X-ray performed for one week's history of dorsal foot pain following an increase in activity. The imaging was performed for investigation of possible stress fracture. The initial X-ray showed abnormal articulation between the base of the third and fourth metatarsals with concave scalloping of the medial margin of the fourth metatarsals, best appreciated on oblique projection (Figures 1(a) and 1(b)). No fracture was identified.

MRI (Figures 1(c)–1(e)) revealed fibrocartilaginous coalition between the third and fourth metatarsals with localized marrow oedema signal and surrounding reactive bone formation.

There is, in addition, a third intermetatarsal bursal-neuroma complex (Figure 1(f)) and low-grade marrow oedema at the plantar border of the third metatarsal head (Figure 1(g)). Symptoms were managed with conservative management and orthotics with the plan to refer for foot and ankle surgical opinion should conservative management fail.

### 2.2. Case 2

**Figure 2:**
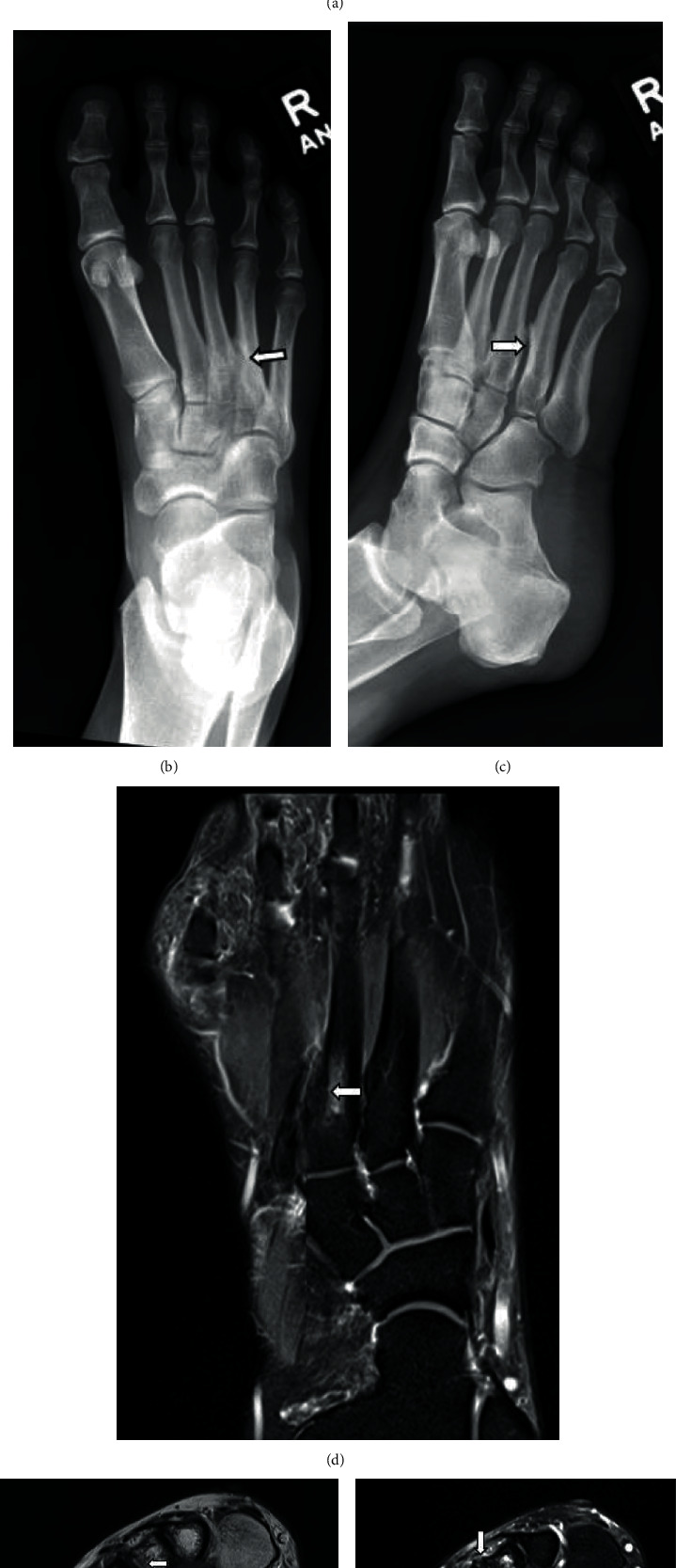
29-year-old female with dorsolateral foot pain. Ultrasound in axial view of the proximal metatarsals (a) shows abnormal bony overgrowth in the dorsal border of the third and fourth intermetatarsal space. This is associated with adventitial bursal formation, soft tissue oedema, and increased vascularity. AP (b) and oblique (c) radiographs show abnormal articulation at the base of the third and fourth metatarsals with sclerosis at the site of the approximation. Coronal PDFS (d) with nonosseous coalition at the third and fourth intermetatarsal spaces. Associated periostitis and intramedullary oedema signal. Axial PD (e) and PDFS (f). Marrow oedema signal. Osteitis and periostitis. Abnormal articulation at this level with surrounding bone spurring.

A 29-year-old female patient underwent radiologic investigation following 3 years' history of dorsolateral forefoot and midfoot pain exacerbated by activity. Initial ultrasound examination revealed dorsal surface metatarsal bony irregularity and adjacent soft tissue oedema and hyperaemia at the region of interest, corresponding to the base of the third and fourth metatarsals (Figure 2(a)). X-ray confirmed the presence of abnormal articulation between these two bases (Figures 2(b) and 2(c)).

MRI revealed nonosseous coalition at the base of the third and fourth metatarsals with localized marrow oedema in the third metatarsal, surrounding periostitis and reactive bony changes including osteophyte lipping across this articulation (Figures 2(d)–2(f)).

A conservative approach was also undertaken, with good results.

## 3. Discussion

**Figure 3:**
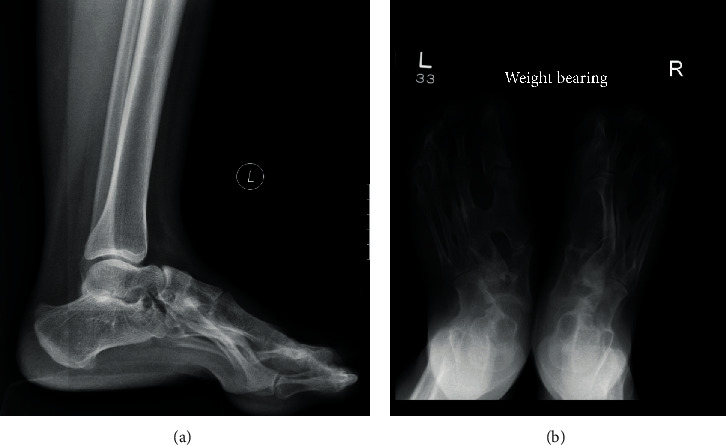
Companion case. 30-year-old man with the Apert syndrome. Lateral (a) and frontal (b) foot X-ray with multiple osseous synostosis involving both feet, including intermetatarsal coalition.

Coalition is defined as abnormal bridging between two bones [1]. The connection can be osseous, fibrous, or cartilaginous. Congenital coalition is a result of failure of mesenchymal differentiation and separation during embryogenesis, resulting in failure of joint formation [2, 3]. This can be an isolated finding or associated with various congenital anomalies such as in the case of the Apert syndrome [11] (Figures 3(a) and 3(b)). In isolated cases, a genetic component with autosomal dominant mode of inheritance with variable penetrance has been proposed [1, 12]. Coalition can also be noncongenital or acquired, such as in cases of trauma or infection [4].

Coalition was classically thought to be present in approximately 1% of the population, although more recent studies have suggested prevalence as high as 13%, given the increased accessibility to imaging for detection of the asymptomatic cases [1]. In the foot, calcaneonavicular and talocalcaneal coalition is the most prevalent, with 90% of foot coalitions involving the calcaneus [2].

Intermetatarsal coalition is much less well known and only a small number of cases have been reported in the English literature (Table 1). For the nonsyndromic congenital coalition, only 8 cases have been reported with the majority (5 case reports) being osseous coalitions [2, 6, 7, 9, 10]. The majority of the coalitions occur in between the 1st and 2nd or the 4th and 5th metatarsals. Only one paper has reported third and fourth metatarsal coalition at the base [3]. All of these are available only in the orthopedic literature; none in radiology literature. These cases are predominantly diagnosed on plain radiographs. Only one case report had CT performed [3] and only a single separate case featured MRI, and this was only performed due to initial suspicion of the radiographic abnormality being secondary to an osteochondroma [8].

The common presentations described are nonspecific dorsolateral or forefoot pain corresponding to the site of the coalition. This is similar to how our cases presented. The cause for the pain is thought to be due to the altered weight-bearing mechanics, with reduced mobility and flexibility as a result of the coalition which then results in inefficient weight distribution between the metatarsal heads and hence increased stress on the forefoot [6]. The sites of increased stress loading would be the plantar borders of the metatarsal heads and the site of the pseudoarthrosis [6], hence explaining the marrow-oedema signal in these locations in our patients (Figure 1(g)). Symptoms typically present in adolescents, likely corresponding to the ossification process of coalition, lending to increased stress and microfractures [3, 12]. Given the increased stress in metatarsal heads, bursal-neuroma complexes in intermetatarsal spaces can also complicate intermetatarsal base coalition [8] (Figure 1(f)).

Radiographs are usually the first imaging modality for investigation of foot pain given the availability and relative low cost. Osseous bridging or hypertrophy of the metatarsals can be readily apparent but is frequently not recognized by the reporting radiologists due to the rarity of the condition. One can be alerted to nonosseous coalition by observing osseous deformity along the margins of the coalition [1]. Other features to look for are additional facets or abnormal articulation in the metatarsals. Features of reactive bone changes or degeneration such as sclerosis, subchondral cyst, and marginal osteophyte formation in an unusual location should also increase a radiologist's alertness to a potential coalition, particularly if present in a younger demographic. Radiographs also provide useful information with regard to weight-bearing alignment.

**Figure 4:**
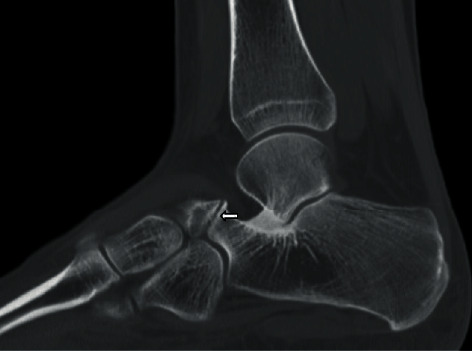
Sagittal hindfoot in a 20-year-old female demonstrating a nonosseous calcaneonavicular coalition. Note the irregular, opposing joint margins.

Computed tomography (CT) with thin slices and 3-dimensional reconstruction ability via surface rendering offers a better appreciation of the anatomy depicted on radiographs without the overlapping composite shadowing. Osseous coalition is defined by its osseous continuity between normally separate structures. Nonosseous coalition can be differentiated from normal joints by its irregular opposing margins, subchondral cyst formation, and usually narrowed space [13] (Figure 4). CT also allows for better characterization of the dimension or size of the coalition, and any secondary bony changes or arthrosis which would aid in surgical decision-making and planning [3].

Ultrasound, whilst of limited value in the diagnosis of coalition, is useful in localizing the pathology and in assessing for secondary inflammatory changes associated with coalition as a potential cause for pain (Figure 2(a)). Not infrequently, this may be the initial imaging modality (by virtue of the referral); thus, the radiologists need a high index of suspicion for this condition. In our case, it also identified a bursal-neuroma complex in the intermetatarsal space (not shown).

Magnetic resonance imaging (MRI) findings are relatively similar to CT with the addition of being able to distinguish between fibrous and cartilaginous coalition and visualization of bone marrow oedema signal. Cartilaginous coalition is T2 hyperintense, and T1 isointense to muscle, whilst fibrous coalition is T2 and T1 iso-hypointense. There are often overlapping imaging features; hence, for simplicity, these can be termed fibrocartilaginous coalition. MRI can also demonstrate bone marrow oedema signal or any stress response associated with the coalition more elegantly, to confirm the coalition being a pain generator.

Other potential soft tissue complications of coalition to assess for, apart from interdigital bursal-neuroma complexes as mentioned above, are plantar plate degeneration and metatarsal head oedema from the altered weight-bearing dynamics [8].

Technetium-99m with single-photon-emission tomography (SPECT) may also show increased uptake at the site of coalition if there is active stress or inflammatory response. None of our patients have undergone any functional imaging.

The treatments described in the literature for the treatment of symptomatic metatarsal coalition include the conservative or surgical approach. The primary aim of the treatment is to correct the foot deformity for better anatomic alignment and function. In general, surgery is usually reserved for cases recalcitrant to conservative management and usually involves resection or disconnection of the coalition [3]. There is also a potential role for diagnostic and therapeutic ultrasound-guided cortisone injection at the site of nonosseous coalition.

In conclusion, intermetatarsal coalition is rarely described, but it is almost certainly an underrecognized condition, particularly in the radiology community. This report adds to the literature by presenting two different cases of intermetatarsal coalitions and describing the imaging findings of different imaging modalities. Potential downstream complications of intermetatarsal coalition are also discussed. Prompt diagnosis of coalition for patients presenting with nonspecific metatarsalgia is important as this condition can be debilitating but treatable and when addressed early, progression to degeneration can be halted. Potential future research includes more large-scale radiology studies to determine the prevalence of the isolated, nonsyndromic intermetatarsal coalition.

## Figures and Tables

**Table 1:** Available literature on intermetatarsal coalition on literature search via PubMed. Keyword coalition or synchondrosis or synostosis AND intermetatarsal or metatarsal.

Primary author/reference	Metatarsals involved and location of coalition	Type of coalition
Cordoba-Fernandez A [2]	4th-5th base	Osseous
Dunn KW [3]	3rd-4th base	Nonosseous
Kachuk KB [5]	4th-5th base	Not applicable
Novak EJ [6]	1st-2nd base	Osseous
Russell N [4]	3rd-4th distal	Nonosseous
Vun SH [7]	4th-5th distal	Osseous
Yang C [8]	1st-2nd base	Nonosseous
Mohammed F [10]	1st-2nd base	Osseous
Aspros D [9]	4th-5th distal	Osseous

## Data Availability

Not applicable
